# Phase Transition of Ice at High Pressures and Low Temperatures

**DOI:** 10.3390/molecules25030486

**Published:** 2020-01-23

**Authors:** Jinjin Xu, Jinfeng Liu, Jinyun Liu, Wenxin Hu, Xiao He, Jinjin Li

**Affiliations:** 1 Shanghai Engineering Research Center of Molecular Therapeutics and New Drug Development, School of Chemistry and Molecular Engineering, East China Normal University, Shanghai 200062, China; xujinjin_stu22@163.com; 2 National Key Laboratory of Science and Technology on Micro/Nano Fabrication, Key laboratory for Thin Film and Microfabrication of the Ministry of Education, Department of Micro/Nano-electronics, Shanghai Jiao Tong University, Shanghai 200240, China; 3 State Key Laboratory of Natural Medicines, Department of Basic Medicine and Clinical Pharmacy, China Pharmaceutical University, Nanjing 210009, China; liujinfeng.1119@163.com; 4 Key Laboratory of Functional Molecular Solids of the Ministry of Education, Anhui Laboratory of Molecule-Based Materials, College of Chemistry and Materials Science, Anhui Normal University, Wuhu 241000, China; 5 The Computer Center, School of Computer Science and Software Engineering, East China Normal University, Shanghai 200062, China; wxhu@cc.ecnu.edu.cn; 6 NYU-ECNU Center for Computational Chemistry at NYU Shanghai, Shanghai 200062, China

**Keywords:** ice phase transition, ice IX, ice XIII, MP2 theory, Raman spectra

## Abstract

The behavior of ice under extreme conditions undergoes the change of intermolecular binding patterns and leads to the structural phase transitions, which are needed for modeling the convection and internal structure of the giant planets and moons of the solar system as well as H2O-rich exoplanets. Such extreme conditions limit the structural explorations in laboratory but open a door for the theoretical study. The ice phases IX and XIII are located in the high pressure and low temperature region of the phase diagram. However, to the best of our knowledge, the phase transition boundary between these two phases is still not clear. In this work, based on the second-order Møller–Plesset perturbation (MP2) theory, we theoretically investigate the ice phases IX and XIII and predict their structures, vibrational spectra and Gibbs free energies at various extreme conditions, and for the first time confirm that the phase transition from ice IX to XIII can occur around 0.30 GPa and 154 K. The proposed work, taking into account the many-body electrostatic effect and the dispersion interactions from the first principles, opens up the possibility of completing the ice phase diagram and provides an efficient method to explore new phases of molecular crystals.

## 1. Introduction

Water is the most studied material on earth and consists of two hydrogen atoms that are attached to a single oxygen atom. The ice phase diagram shows the preferred physical state under different thermodynamic variables, with the small changes in temperature or pressure causing the structural change from one physical state to another [1,2,3]. It is well-known that more than 18 crystal ice phases from ice I to ice XVII and superionic phase (XVIII) [4,5,6]. At least three amorphous ice forms have been observed in laboratory at gigapascal pressures and the temperature range below 0 degrees Celsius. However, there are still some undetermined phase transition boundaries in the areas of high pressure and low temperature, such as that between ice IX and ice XIII. Ice IX was first observed, and it was converted from ice III through cooling [7]. Some experiments have found that ice V can be converted to ice XIII under certain conditions [8]. Other studies also found that ice IX and ice V have a relationship of mutual transformation under certain circumstances [9]. By analyzing the phase diagrams, this connection reminds us of conversion between Ice IX and Ice XIII, but the relationship between them has not yet been determined. In addition, from the experiments, the researchers have measured the crystal structures of ices IX and XIII and their respective vibrational spectra, but the phase boundary between ices IX and XIII is still undetermined in the phase diagram [4,7,8,9]. Therefore, the structural characteristics of ices IX and XIII at high pressure and ultralow temperature are extremely important.

Exploring molecular structures in extreme environments have many limitations in laboratory, such as the weak of experimental signal, the harsh conditions of high-pressure equipment, etc., but effective theoretical predictions provide an alternative approach to study the detailed crystal structures. Based on the second-order Møller–Plesset perturbation (MP2) theory, the present work proposes an ab initio method to investigate the structures, equation of state, Raman spectra and Gibbs free energies of ices IX and XIII, and predicts their phase transition at high pressure and low temperature, which provides opportunities to advance the development of high pressure structural determination through computation [10].

With the continuous increase of computer power, molecular simulation becomes a powerful tool to predict different molecular characteristics. The ability of predictive computer simulation depends on the accuracy of the potential energy calculation [11]. Both classical and quantum simulation methods have been previously performed on the calculations of ice structures and phase transitions [12,13]. The classical methods rely on the empirical force fields, such as TIP3P, TIP4P, TIP5P, and SPC/E [13,14,15,16,17,18], which have the transferability restrictions owing to the lack of electronic polarization [19] and ultimately lead to the inaccurate prediction. The quantum mechanical (QM) methods, include the density-functional theory (DFT), which overestimated the phase transition boundary [20,21], and other high-level wavefunction theories, such as the MP2 method [22,23,24,25]. When calculating the phase transition at high pressures, the dispersion energy is not well described by the DFT method, which tends to overestimate the hydrogen bonding interactions and substantially decreases the prediction accuracy [26]. For example, the ice XV was predicted to be a ferroelectric structure with Cc space group symmetry based on the DFT method [27,28], while the experimental measurement determined that the crystal structure of ice XV was antiferroelectric with a $P\bar{1}$ space group symmetry [28,29,30]. The proton ordering in phase XV has been extensively studied by high-level wavefunction theories, such as MP2 and coupled cluster theory (CCSD(T)), which explains the structure of ice XV [29,30,31]. It is worth noting that the previous studies [30,31] using the MP2 method give different conclusions on the ground state of ice XV (ref. 30 agrees with the experimental results; whereas ref. 31 agrees with prior DFT results and discusses a possible boundary condition resolution). Therefore, the prediction of the small energy differences between various phases of ice usually require accurate computational approaches. The high-precision calculation method has prompted us to study the transformation between different phases of ice from high-level wavefunction theories, and to predict the ice structure and phase diagram under extreme conditions.

In this work, we demonstrate the practicability of MP2 theory by applying it to the structure simulation of ice phases IX and XIII, along with the electrostatically embedded generalized molecular fractionation (EE-GMF) method [32,33]. Due to the limitation of computation time, the high-level quantum chemistry methods, such as the MP2 theory, cannot be directly applied for calculations of macromolecules. The EE-GMF is a fragment-based quantum mechanical (QM) method [24,34,35,36,37], which divides the internal energy per unit cell of the crystal into a proper combination of the energies of monomers and overlapping dimers, and therefore can treat the molecular crystals at the QM level efficiently. The fragmented monomers and dimers are embedded in the electrostatic field of the rest of the crystalline environment [22,23,24,35,36,38]. The embedding field is essential and, in our method, consists of self-consistently determined atomic charges at the Hartree–Fock level. In addition, the interaction energy between two fragments within a distance threshold is calculated by QM, while the interaction between two long-range interacting fragments is treated by charge-charge Coulomb interaction. Based on the EE-GMF-MP2 method, the predicted structures, and vibrational spectra of ice phases IX and XIII are in good agreement with the experiment. The predicted transition from ice IX to ice XIII occurs at 0.30 GPa and 154 K, with the transition pressure decreasing slightly as the increase of temperature.

## 2. Results and Discussion

### 2.1. Crystal Structures

Ice phase IX is an ultralow temperature solid structure, which is stable at temperatures below 140 K and pressures between 0.2 and 0.4 GPa. It has a square lattice with a density of 1.16 g/cm^3^, and can be converted to ice II by warming. Ice phase IX has a similar structure with ice III, except that ice IX is a proton ordered structure while ice III is a proton disordered structure [39,40]. Furthermore, ice phase XIII is a monoclinic, metastable, and proton ordered structure, which is formed by doping ice V below 130 K and at 0.5 GPa to facilitate the phase transition [8,41]. Therefore, ice phase XIII structure has a lower temperature range than ice IX. The selected lattice parameters for ice IX are a = b = 6.692 Å, c = 6.715 Å; α = β = γ = 90° [29], while the lattice parameters for ice XIII are a = 9.2417 Å b = 7.4724 Å, c = 10.2970 Å, β = 109.6873° [7].

**Figure 1:**
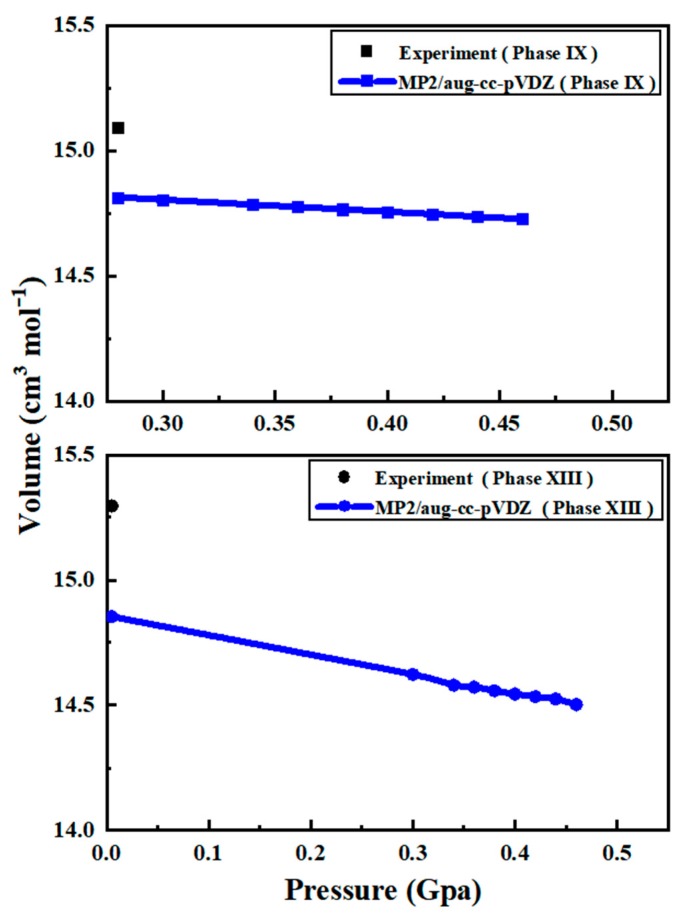
The calculated volumes of ice phases IX (a) and XIII (b) as a function of pressure, along with the experimental data which are taken from Ref. [7] (phase IX) and Ref. [8] (phase XIII).

Figure 1 shows the predicted volumes of ice phases IX (a) and XIII (b), where the black dots denote the observed data while the blue curves are the calculated volumes by the EE-GMF-MP2/aug-cc-pVDZ method. Comparing with the first principles calculation of ice structures at the HF level, which predicted too short O-H bond and too long O···H distance owing to the underestimation of hydrogen bond energy [42,43,44,45], the MP2 theory eliminates this drawback through inclusion of the electronic correlation and predicts reasonable results. As shown in Figure 1, the predicted volumes for phases IX and XIII experience volume decreases with the increase of pressure, with no discontinuities or any other anomalies within 0–0.5 GPa. For ice phases IX and XIII, the MP2 theory is accurate in predicting the lattice constants *a* and *b*, but has an error of 0.03 to 0.1 Å when predicting *c* due to the strong hydrogen bonding interaction along *c* direction. Such errors lead to the slight volume difference between the calculated results and observed data, as shown in Figure 1. The comparison of calculated and observed lattice constants of ice phases IX and XIII are plotted in Figures S2 and S6 of the Supplementary Materials (SM).

### 2.2. Vibrational Spectra

**Figure 2:**
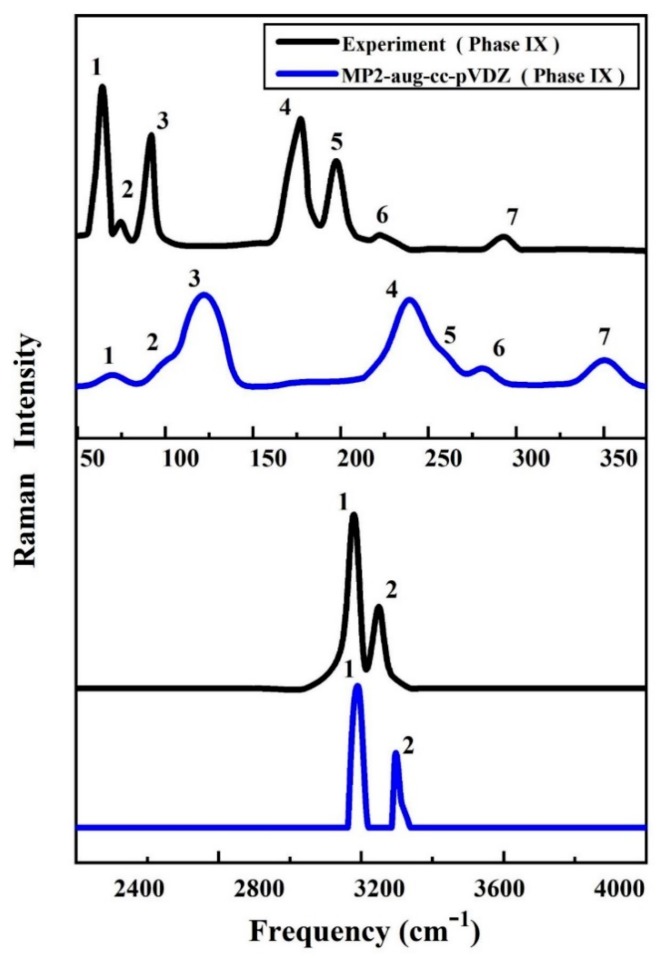
The calculated and observed Raman spectra of ice phase IX in liberational frequency (low frequency) and stretching frequency (high frequency) regions at 0.28 GPa. The calculation is carried out using EE-GMF-MP2/aug-cc-pVDZ, while the observed Raman spectra are taken from Ref. [46,47,48,49].

**Figure 3:**
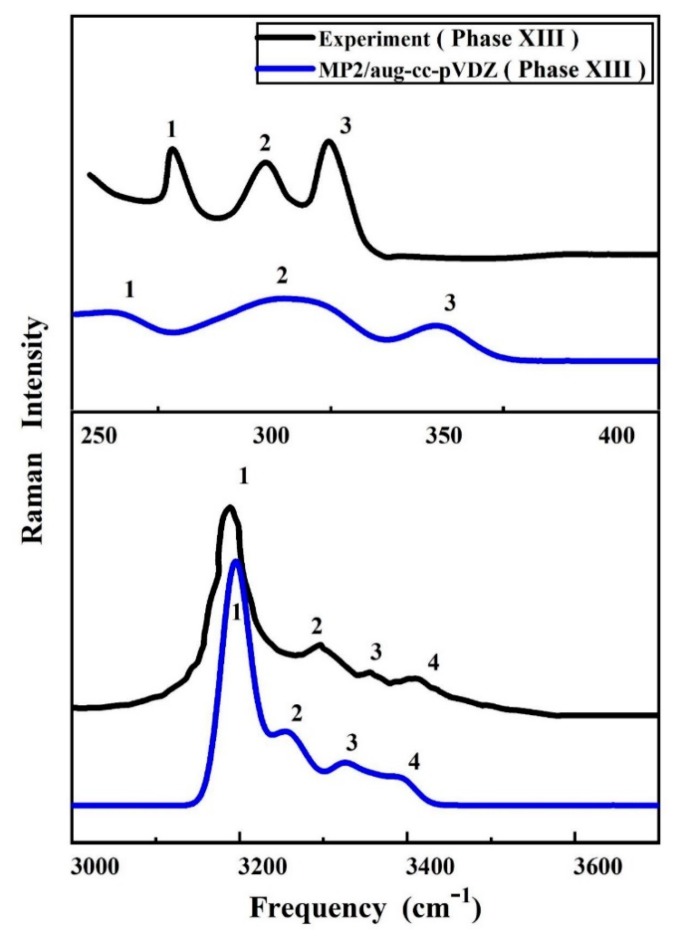
The calculated and observed [50] Raman spectra of ice phase XIII at 10 MPa. The calculated spectra are performed using the EE-GMF-MP2/aug-cc-pVDZ method.

The vibrational spectrum is regarded as a chemical recognition of a particular molecule or material, which can be used to quickly identify or distinguish structures. Figure 2 and Figure 3 show the calculated Raman spectra of ice phases IX and XIII using the EE-GMF-MP2/aug-cc-pVDZ method, along with the experimental data. In Figure 2, the MP2 theory predicted 7 Raman bands in the low frequency region, which are assigned to 7 observed Raman peaks from 50 to 350 cm^−1^. From the results of experiment and theory, the numbers of Raman peaks from theoretical simulation and experiment are the same. For the three Raman peaks below 150 cm^−1^, the deviations between the experimental values and theoretical results are relatively small, and the maximum deviation of the theoretical prediction is about 30 cm^−1^ with reference to the experimental observation for the third peak. There are three experimental Raman peaks within the frequency region between 150 and 350 cm^−1^. Among these three peaks, the deviations of theoretical predictions are between 50 to 70 cm^−1^ as compared to the experimental results. Therefore, our theoretical calculations qualitatively reproduced the experimental Raman spectrum of ice phase IX in the low frequency region. Furthermore, for the high frequency region, the predicted 2 discernible Raman bands of ice phase IX are assigned to 2 observed Raman peaks from 2800 to 3600 cm^−1^. Figure 3 shows the calculated and observed Raman bands of ice phase XIII at 10 MPa, with 3 and 4 Raman bands at low frequency and high frequency regions, respectively. The half-widths at half-maximum (HWHM) for Lorentz broadening were set to 20 and 8 cm^−1^ in the high frequency region and low frequency region, respectively. The consistency of Raman peak positions between the MP2 theory and experiment is quantitative and consistent, indicating that the MP2 method can provide accurate prediction of the ice structures at high pressures.

**Figure 4:**
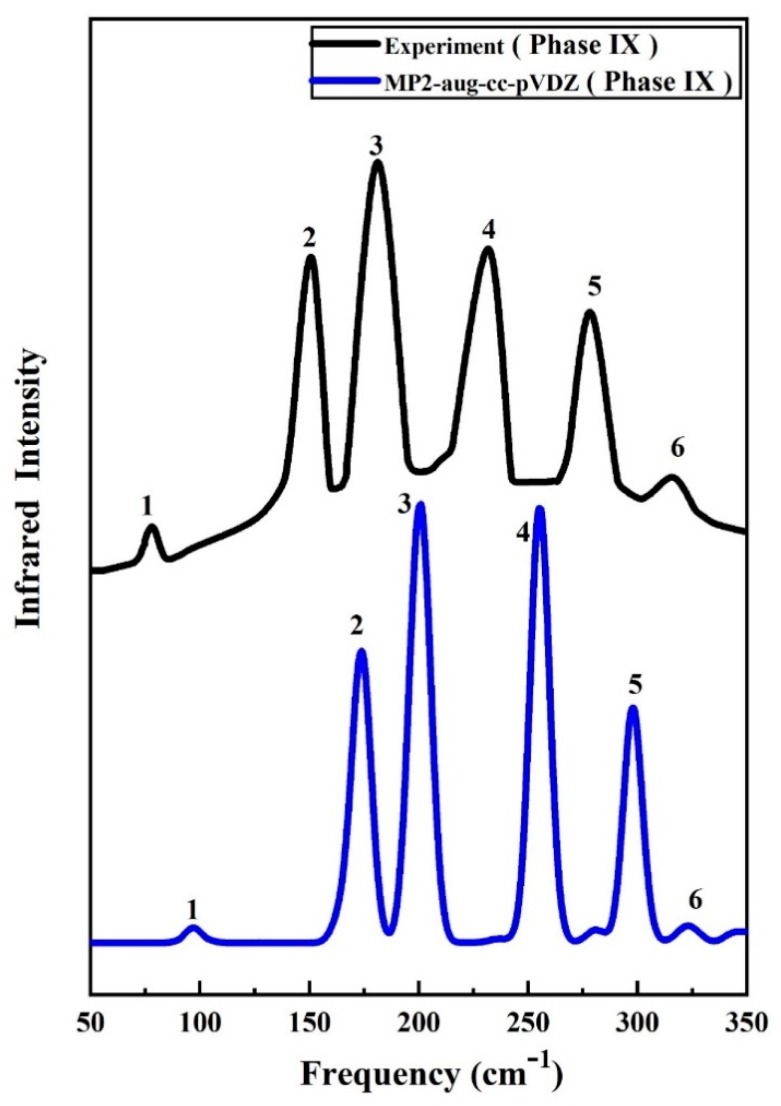
The calculated infrared (IR) spectrum of ice IX at 0.28 GPa, along with the experimental data taken from Ref. [46].

The proposed ab initio method allows us to further calculate the vibrational IR spectra of ice phases IX and XIII. Figure 4 shows the IR spectra comparison between the experiment and calculated results by EE-GMF-MP2/aug-cc-pVDZ. As one can see from Figure 4, there are six predicted IR peaks for ice phase IX, with a half-width at half-maximum (HWHM) of 8 cm^−1^ for each band. The agreement of IR spectra between the experiment and theory further confirms the accuracy of the proposed ab initio method and provides an approach to identify the structural phases for molecular crystal. So far, no experimental IR work has been carried out and reported for ice phase XIII, and thus the present work only reports the IR comparison for ice IX. We hope to carry out further Raman and IR spectral experiments in the future to verify the proposed spectral prediction. More Raman and IR spectra analysis for ice phases IX and XIII can be found in the Supplementary Materials.

### 2.3. Phase Transition

**Figure 5:**
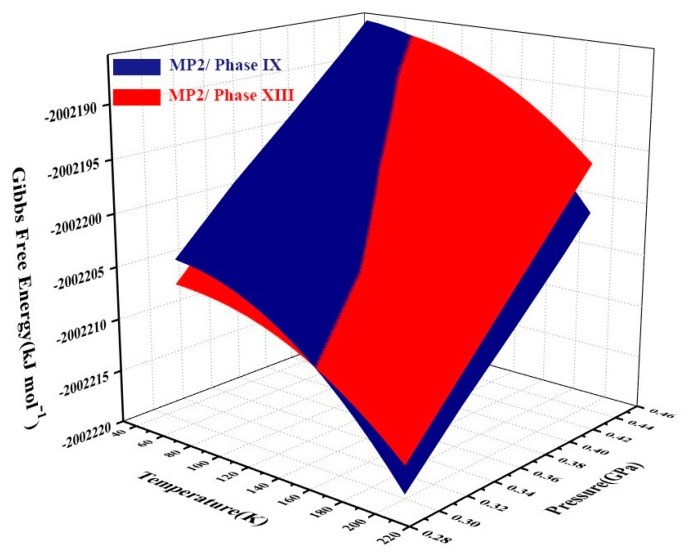
Gibbs free energy surfaces of phases XIII (red) and IX (blue) from 0.30 to 0.46 GPa and within the range of 40–200 K. The intersection of red and navy surfaces denotes the phase transition boundary between phase XIII and phase IX, calculated by EE-GMF-MP2/aug-cc-pVDZ.

**Figure 6:**
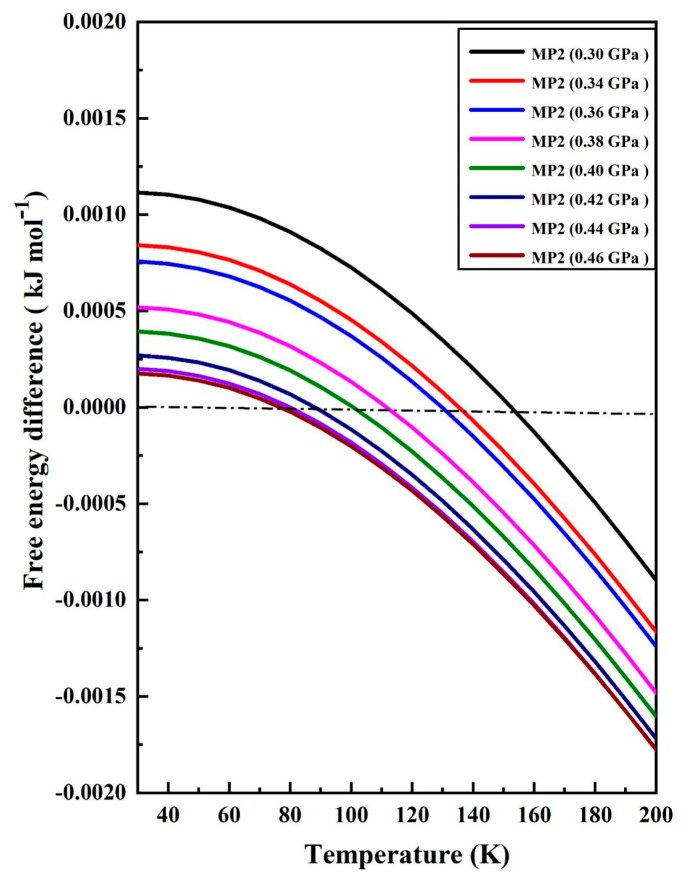
The Gibbs free energy difference between ice phases IX and XIII as a function of temperature under different pressures. The positive values indicate that the phase XIII is more stable than phase IX.

The Gibbs free energies of ice phases IX and XIII are further calculated by the EE-GMF method. Figure 5 plots the free energy surfaces of phases IX and XIII as functions of temperature and pressure. Figure 6 shows the temperature dependence of the Gibbs free energy difference between phases IX and XIII at different pressures. As shown in Figure 5, in the low temperature region, the phase XIII (red) has a lower free energy and its structure is more stable than phase IX. At high temperatures, the structure of phase IX is more stable under high pressure. The ice phase diagram can be obtained by calculating the Gibbs free energy.

Figure 6 shows the Gibbs free energy difference between ice phases IX and XIII, where the zero difference experiences temperature decrease with the increasing of pressure [51]. For example, at the pressures of 0.30, 0.34, 0.36, 0.38, 0.40, 0.42, 0.44, and 0.46 GPa, the zero differences of Gibbs free energies between phases IX and XIII occur at the temperatures of 154, 136, 126, 118, 109, 88, 76, and 74 K, respectively. The effect of transition temperature decreasing as the increase of pressure can be traced back to the hardening of acoustic phonons under high pressures.

**Figure 7:**
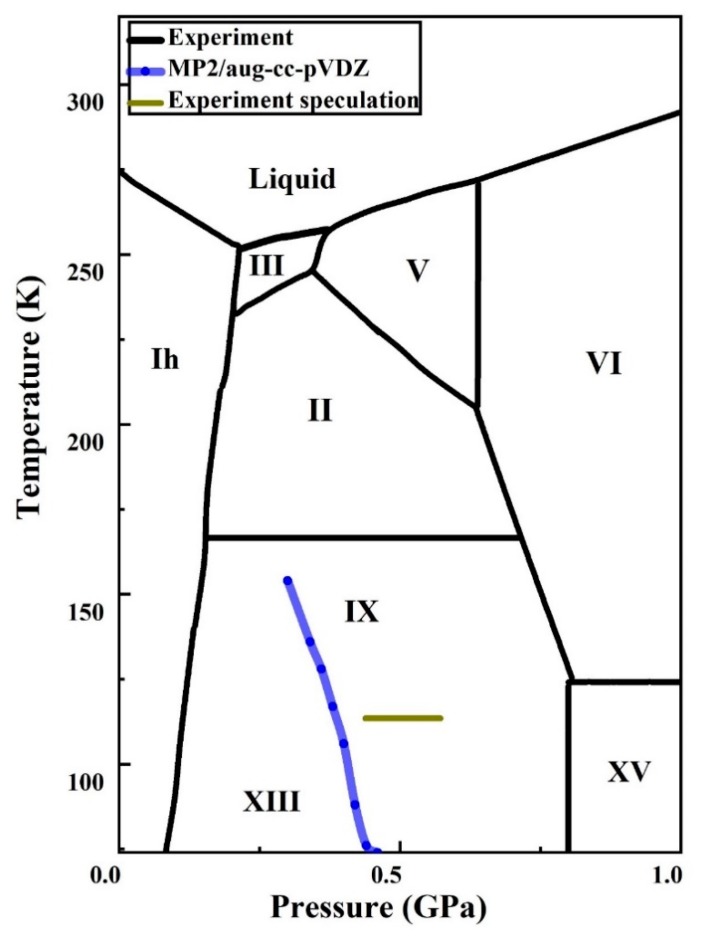
The phase diagram of ice. The black curves represent experimental data [7,8,9,29,39,54,55,56,57,58,59], while the blue and gray curves denote the calculated and speculated experimental [8,9,29] phase boundary between ice phase IX and phase XIII. The solid lines drawn here do not explicitly indicate regions of stability of all the ice phases. Several of these boundaries correspond to extrapolated equilibrium lines from experiments.

Figure 7 shows the ice phase transition with various temperatures and pressures. The blue curve is the predicted phase boundary by EE-GMF-MP2/aug-cc-pVDZ, indicating that the phase transition between phases IX and XIII occurs at 0.46 GPa and 74 K, and slightly decreases to 0.44 GPa at 76 K, 0.42 GPa at 88 K, 0.40 GPa at 109 K, 0.38 GPa at 118 K, 0.36 GPa at 126 K, 0.34 GPa at 136 K and 0.30 GPa at 154 K, respectively. Ice phases IX and XIII are the least studied structures in laboratory compared to other phases, owing to their ultralow temperatures. The predicted phase transition between IX and XIII by MP2 has not been verified in laboratory, but pioneers speculated that such phase transition would take place around temperature of 113 K and pressure range of 0.43–0.63 GPa (the gray line in Figure 7) [8,9,52,53]. Therefore, the proposed theoretical prediction of phase transition between phases IX and XIII is in good agreement with the experiment and close to the temperature and pressure range of the experimental speculation.

In this study, we mainly focused on the Gibbs free energy differences between Ices IX and XIII in the high pressure and low temperature region. This work demonstrates the relative stability between Ices IX and XIII in terms of Gibbs free energy. In the future study, the other phases, such as Ices II, Ih and XV, under low temperatures may also be considered and thus we could provide a more comprehensive comparison of relative stabilities of those phases in the high pressure and low temperature region. The researches along these lines are underway in our laboratory.

### 2.4. Implementation

The distance threshold was set to 5.0 Å for determining fragment pairs used in quantum mechanical calculations. The interactions beyond the distance threshold were treated by classical Coulomb interactions. All QM calculations were carried out using the Gaussian09 program [60].

### 2.5. Limitations

The current EE-GMF-MP2/aug-cc-pVDZ calculations may have errors arising from the incompleteness of the base set [61,62]. The harmonic approximation in phonon calculations is more appropriate for Gibbs free energy predictions of molecular crystals at low temperatures [63]. The EE-GMF scheme for molecular crystals is truncated at the two-body QM interactions, while the three-body and higher-order many-body QM interactions are implicitly incorporated in the electrostatic embedding scheme. For accurate description of the intermolecular QM interaction in molecular crystals, three-body QM interactions sometimes have non-negligible contributions, and thus need to be treated explicitly.

## 3. Methods

**Figure 8:**
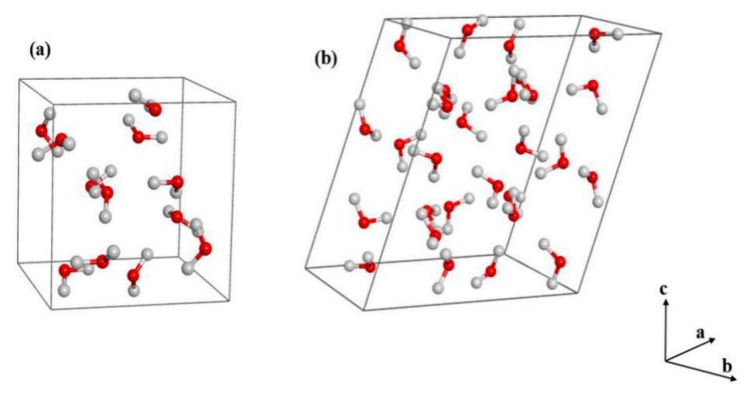
The crystal structures of ice IX (**a**) and XIII (**b**). The red and grey spheres represent the oxygen and hydrogen atoms, respectively.

The crystal structures, vibrational spectra, Gibbs free energies and phase transition of ice phases IX and XIII were calculated at the MP2 level with the aug-cc-pVDZ basis set [25,63,64]. The EE-GMF method was used to calculate the internal energies, where the effects of pressure were included in the geometric optimization. Based on the EE-GMF method, the monomers and dimers in the 3 × 3 × 3 unit cells were treated quantum mechanically in the embedding electrostatic field of 11 × 11 × 11 unit cells, while the long-range electrostatic contribution was treated in a 41 × 41 × 41 supercell. Figure 8 shows the unit cells of ice phases IX and XIII, where the unit cell of ice IX contains 12 molecules with a space group of P4_1_2_1_2, and the ice XIII has 28 molecules in the unit cell with a symmetry of P2_1_/a.

The electronic enthalpy per unit cell ($H_{u\_\mathrm{cell}}$) of a three dimensional, periodic molecular crystal is approximated accurately by

(1) $$H_{u\_\mathrm{cell}}=\sum_{k}E_{k(0)}+\frac{1}{2}\sum_{i}\sum_{k,l}\left\{E_{k(0)l(i)}-\;E_{k(0)}-\;E_{l(i)}\right\}+E_{\mathrm{LR}}+PV$$

where the energy of the *k*th monomer in the central unit cell (*0*th) is *E_k_*_(0)_, and the energy of the dimer composed of the *k*th monomer in the central unit cell and the *l*th monomer in the *i*th unit cell is *E_k_*_(0)*l*(i)_. The three integers of the specified cell are represented by *i*. The remote electrostatic energy correction is *E*_LR_ (i.e., the long-range part of the Madelung constant), the pressure is *P*, and the unit cell volume is *V*. It should be noted that being excluded from the second sum is *k* = *l* and *i = 0*. Although the truncation of multibody expansion after the two-body terms (dimers) achieves an excellent cost-accuracy balance, the series can also be truncated after a three-body or higher-order term.

The first and second derivatives of enthalpy in Equation (1) for atomic coordinates are related to atomic force and force constant, respectively. Derivatives relating to lattice constants give lattice forces as follows,

(2) $$\frac{\partial H_{u\_\mathrm{cell}}}{\partial x}=\sum_{i}\frac{\partial E_{i(0)}}{\partial x}+\frac{1}{2}\sum_{n}\sum_{i,j}\left\{\frac{\partial E_{i(0)j(n)}}{\partial x}-\frac{\partial E_{i(0)}}{\partial x}-\frac{\partial E_{j(n)}}{\partial x}\right\}+\frac{\partial E_{LR}}{\partial x}$$

(3) $$\frac{\partial^{2}H_{u\_\mathrm{cell}}}{\partial x\partial y}=\sum_{i}\frac{\partial^{2}E_{i(0)}}{\partial x\partial y}+\frac{1}{2}\sum_{n}\sum_{i,j}\left\{\frac{\partial^{2}E_{i(0)j(n)}}{\partial x\partial y}-\frac{\partial^{2}E_{i(0)}}{\partial x\partial y}-\frac{\partial^{2}E_{j(n)}}{\partial x\partial y}\right\}+\frac{\partial^{2}E_{LR}}{\partial x\partial y}$$

(4) $$\frac{\partial H_{u\_\mathrm{cell}}}{\partial a}=\frac{1}{2}\sum_{n}\sum_{i,j}\left\{\frac{\partial E_{i(0)j(n)}}{\partial a}-\frac{\partial E_{j(n)}}{\partial a}\right\}+\frac{\partial E_{LR}}{\partial a}+P\frac{\partial V}{\partial a}$$

where *a* is a lattice constant, *x* and *y* can be collective in-phase coordinates (for balanced geometric determination) or single atomic coordinates. It should be noted that these equations ignore the derivative of the embedded electrostatic field. The geometric derivatives of dipole moment and polarizability can also be calculated using the fragmentation approach and provide information of infrared (IR) and Raman intensity.

We have included the background charges in the 11 × 11 × 11 supercell. The last term of Equation (1), gives the long-range interaction in the 41 × 41 × 41 supercell through the charge-charge Coulomb interaction,

(5) $$E_{\mathrm{LR}}=\frac{1}{2}\sum_{|n|>s}^{L}\sum_{k,l}\sum_{\gamma ,\eta }\frac{q_{k(0)}^{\gamma }q_{l(n)}^{\eta }}{R_{k(0)l(n)}^{\gamma \eta }}$$

By calculating the enthalpy of the per unit cell, the effect of pressure is taken into account. The following expressions can be used to calculate IR intensity and Raman intensity,

(6) $$I_{nk}\propto {\left|\frac{\partial \mu_{\mathrm{EE}-\mathrm{GMF}}}{\partial Q_{nk}}\right|}^{2}$$

(7) $$R_{nk}\propto \frac{3}{2}{\left\{\sum_{i=x,y,z}\frac{\partial \alpha_{\mathrm{EE}-\mathrm{GMF}}^{ii}}{\partial Q_{nk}}\right\}}^{2}+\frac{21}{2}{\sum_{i,j=x,y,z}\left\{\frac{\partial \alpha_{\mathrm{EE}-\mathrm{GMF}}^{ij}}{\partial Q_{nk}}\right\}}^{2}$$

where *Q*_*n***k**_ is the corresponding normal mode. Only the zone-center (**k** = **0**) vibrations have nonzero intensities and thus are IR- or Raman-active. In this study, the frequency calculated using MP2/aug-cc-pVDZ is scaled by 0.94. In addition, *μ*_EE−GMF_ is the dipole moment of the central unit, and $\alpha_{\mathrm{EE}-\mathrm{GMF}}^{ij}$ is its polarizability, which can be derived based on the EE-GMF method [63]. The Gibbs free energy per unit cell, $G_{u\_\mathrm{cell}}$, at temperature *T* is obtained by

(8) $$G_{u\_\mathrm{cell}}=H_{u\_\mathrm{cell}}+U_{\nu }-TS_{\nu }$$

where *U*_v_ is the zero-point vibration energy per unit cell at the temperature of *T*, and *S*_v_ is the vibration entropy per unit cell. For molecular crystals with large band gaps, only temperature effects due to phonons need to be considered. Both *U*_v_ and *S*_v_ can be derived from the partition function of phonons, *Z_v_*. In the harmonic approximation and atomic units, *Z_v_* can be written as,

(9) $$Z_{\nu }=\prod_{n}\prod_{k}\frac{e^{-\beta \omega_{nk}/2}}{1-e^{-\beta \omega_{nk}}}$$

where ω_*n***k**_ is the frequency of the phonon with the wave vector **k** in the *n*th phonon branch. The product on **k** must be taken over all *K* evenly spaced **k** grid points in the reciprocal unit cell. In this study, the **k**-grid of 21 × 21 × 21 (K = 9261) is used. According to thermodynamics, we have

(10) $$U_{\nu }=\frac{T}{\beta k}\frac{\partial \ln Z_{\nu }}{\partial T}=\frac{1}{k}\sum_{n}\sum_{k}\omega_{nk}(\frac{1}{2}+\frac{1}{e^{\beta \omega_{nk}}-1})$$

(11) $$S_{\nu }=\frac{1}{\beta TK}\sum_{n}\sum_{k}\left\{\frac{\beta \omega_{nk}}{e^{\beta \omega_{nk}}-1}-\ln(1-e^{-\beta \omega_{nk}})\right\}$$

The right side of these equations can be easily evaluated with phonon dispersion *ω*_*n***k**_.

## 4. Conclusions

From small molecules to macroscopic structures, the phase diagrams of molecular crystals have always been the hot research of scientists. The proposed fragment-based QM calculation using the MP2 method is different from the traditional methods such as the classical force field and DFT theory. The difficulty in phase diagram prediction stems from the high cost of structural screening and the low precision of the applicable method. The proposed MP2 theory combining with the fragmentation approach enables high-precision and efficient calculations on complex systems at finite pressures and temperatures, which can accurately describe all cohesive interactions and obtain reasonable results when predicting structures and energies of molecular structures and produce convinced ice phase diagram via ab initio calculation. Ice phases IX and XIII are two molecular crystals at the high pressure and low temperature, that have rarely been studied by both experiment and theory. In the present work, we quantitatively reproduce their respective structures, and vibrational spectra at the MP2/aug-cc-pVDZ level. The predicted phase transition between the phase IX and phase XIII falls within the temperature and pressure range of the experimental speculation. This work confirms for the first time the positions of phases IX and XIII in ice phase diagram at high pressures and is consistent with the experimental speculation, which can be used to assist in the high pressure exploration of molecular phases with potentially important applications.

## Supplementary Materials

The following are available online, Figure S1: The crystal structure of ice IX., Figure S2: Comparison of calculated and observed lattice constants of ice IX as a function of pressure, Figure S3: The Raman bands of ice phase IX in the low frequency region under different pressures, Figure S4: The pressure dependence of the Raman bands of ice IX in the high frequency region as a function of pressure, Figure S5: The unit cell of ice XIII, Figure S6: Comparison of the calculated and observed lattice constants of ice XIII, Figure S7: The Raman bands of ice XIII in the low frequency region under different pressures, Figure S8: The Raman bands of ice phase XIII in high frequency region under different pressures, Figure S9: The calculated IR spectrum of ice phase XIII under ambient pressure, Table S1: Average computational time of EE-GMF at the MP2/aug-cc-pVDZ level.
