# Supplementary material for: Phase Transition of Ice at High Pressures and Low Temperatures
Source: Molecules. 2020 Jan 23;25(3):486. doi: 10.3390/molecules25030486 (PMC7037513; doi:10.3390/molecules25030486)
Supplement: Supplementary file 1 [file molecules-25-00486-s001.pdf]

Supplementary Materials for

Phase Transition of Ice at High Pressures and low Temperatures

Jinjin Xu[1,2†], Jinfeng liu[3†], Jinyun Liu[4], Wenxin Hu[5], Xiao He[1,6]* and Jinjin Li[2,4]**

[1]*Shanghai Engineering Research Center of Molecular Therapeutics and New Drug Development, School of Chemistry and Molecular Engineering, East China Normal University, Shanghai, 200062, China*

[2]*Key laboratory for Thin Film and Microfabrication of the Ministry of Education, Department of Micro/Nano-electronics, Shanghai Jiao Tong University, Shanghai, 200240, China*

[3]*State Key Laboratory of Natural Medicines, Department of Basic Medicine and Clinical Pharmacy, China Pharmaceutical University, Nanjing, 210009, China*

[4]*Key Laboratory of Functional Molecular Solids of the Ministry of Education, Anhui Laboratory of Molecule-Based Materials, College of Chemistry and Materials Science, Anhui Normal University, Wuhu, Anhui 241000, China*

[5]*The Computer Center, School of Computer Science and Software Engineering, East China Normal University, Shanghai 200062, China*

[6]*NYU-ECNU Center for Computational Chemistry at NYU Shanghai, Shanghai, 200062, China*

[*]Correspondence to: Jinjin Li (lijinjin@sjtu.edu.cn) (J.L.), Xiao He (xiaohe@phy.ecnu.edu.cn) (X.H.) and Jinyun Liu (jyliu@ahnu.edu.cn) (J.L.)

[†]These two authors contributed equally to this work.

In this Supplementary Materials (SM), we show the details of the predicted crystal structures, Raman spectra, IR spectra, and vibrational frequencies of ice phases IX and XIII. Ices IX and XIII are proton ordered structures. Ice IX is a low-temperature equilibrium, slightly denser structure, with a space group of $P4_12_12$ and cell dimensions of a=b=6.692 Å, c=6.715 Å, and α=β=γ=90° at 165 K and 280 MPa. Figure S1 shows the crystal structures of ice IX, where each unit cell contains 12 water molecules [1-3]. Ice XIII can be formed by doping with 10 mM HCl below 130 K and at 500 MPa to facilitate the phase transition. The crystal structure of Ice XIII is shown in Figure S5, where each unit cell contains 28 water molecules. Ice XIII is a monoclinic structure with a space group of $P2_1/a$ and cell dimensions of a=9.24 Å, b=7.47 Å and c=10.30 Å, α=90°, β=109.7° and γ=90° at atmospheric pressure and 80 K [4, 5].

Figures S2 and S6 show the comparisons of calculated and observed lattice constants of ice phases IX and XIII, respectively, where the MP2 results are in good agreement with the observed data. As shown in Figs. S2 and S6, the lattice constants gradually decrease with the increase of pressure, which are consistent with the experiments. Based on the EE-GMF method at the MP2/aug-cc-pVDZ level, Figures S3 and S4 show the calculated and observed Raman frequencies for ice IX [6-8] in the low frequency (from 180 cm^{-1} to 260 cm^{-1}) and high frequency (from 3,050 cm^{-1} to 3,250 cm^{-1}) regions, respectively. As shown in Fig. S3, the Raman vibrational frequency increases gradually with the increase of pressure, while in Fig. S4 the Raman frequency in the high frequency region decreases gradually with the increase of pressure. Figures S7 and S8 show the calculated Raman frequency for ice XIII, where the Raman frequency increases (see Fig. S7) and decreases (see Fig. S8) with the increase of pressure, respectively.

The IR spectral analysis can be found in Figure 5 for ice IX and Figure S9 for ice XIII, respectively. The comparison between calculated and observed IR spectra for ice IX is shown in Fig. 5, where the MP2 calculation quantitatively reproduces the 6 observed IR vibrational frequencies. Furthermore, ice XIII is a low temperature phase with rare vibrational spectra reported in laboratory. Therefore, there are no experimental Raman or IR spectra for comparison in this work. Fig. S9 shows the calculated IR spectrum of ice XIII[4, 9] at ambient pressure with four peaks in the frequency region between 150 and 375 cm^{-1}.

Methods

The details of the EE-GMF method are described in ref. 10. By calculating the enthalpy of the per unit cell, the effect of pressure is taken into account. The dynamical force constant matrix of a periodic system can be expressed as[11-12],

$$\mathrm{D}(r_{\mathrm{A}}, r_{\mathrm{B}}, \mathbf{k}) = \frac{1}{\sqrt{m_{\mathrm{A}} m_{\mathrm{B}}}} \sum_{n=-s}^{s} H(\mathbf{r}_{\mathrm{A},0}, \mathbf{r}_{\mathrm{B},n}) e^{-i\mathbf{kR}(n)} \qquad \text{(S1)}$$

where **k** represents a given point in the Brillouin zone, and $H(\mathbf{r}_{\mathrm{A},0}, \mathbf{r}_{\mathrm{B},n})$ is the second derivative of the total energy per unit cell with respect to atom A in the *0*th cell and atom B in the *n*th cell at the equilibrium geometry. Moreover, m_{A} and m_{B} denote the atomic masses of atoms A and B, respectively. The number of neighboring unit cells for QM treatment was truncated at $S = 2$, and the number of k-points was set to 21 in each of x, y, z dimensions. Once the dynamical force constant matrix of a periodic system is obtained, one can calculate the vibrational frequencies as well as the corresponding normal modes by diagonalizing the matrix $\mathrm{D}(\mathbf{r}_{\mathrm{A}}, \mathbf{r}_{\mathrm{B}}, \mathbf{k})$.

$$\left| D(\mathbf{r}_{\mathrm{A}}, \mathbf{r}_{\mathrm{B}}, \mathbf{k}) \text{-} \omega^2 \mathbf{I} \right| = 0 \qquad \text{(S2)}$$

where ω and **I** represent the vibrational frequency and a unit matrix, respectively. In simulating the infrared (IR) and Raman spectra, only the zone-center ($\mathbf{k} = 0$) vibrations have nonzero intensities and thus are IR- or Raman-active. Therefore, the force-constant matrix $D(\mathbf{0})$ was used for the vibrational frequency calculation of the IR and Raman spectra. Eqs. 6 and 7 are used to calculate the IR and Raman intensities.

1. Ice phase IX

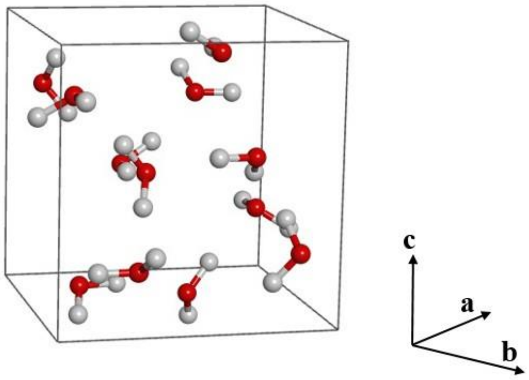

Figure S1. The crystal structure of ice IX, where the unit cell contains 12 molecules[1]. Oxygen atoms are represented by red spheres, and hydrogen atoms are represented by smaller grey spheres.

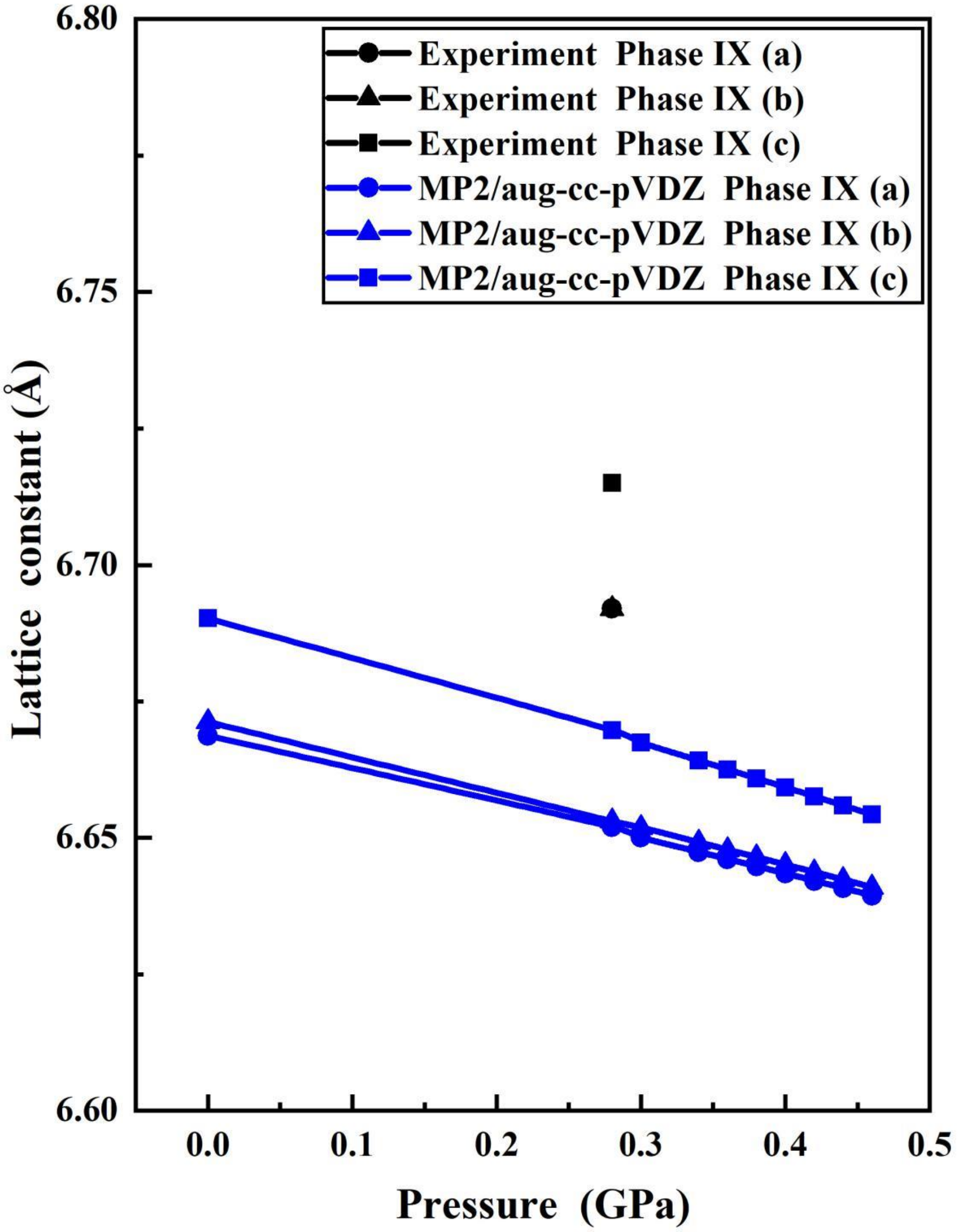

Figure S2. Comparison of calculated and observed lattice constants of ice IX as a function of pressure. The experimental data are taken from the work by Londono et al. [1].

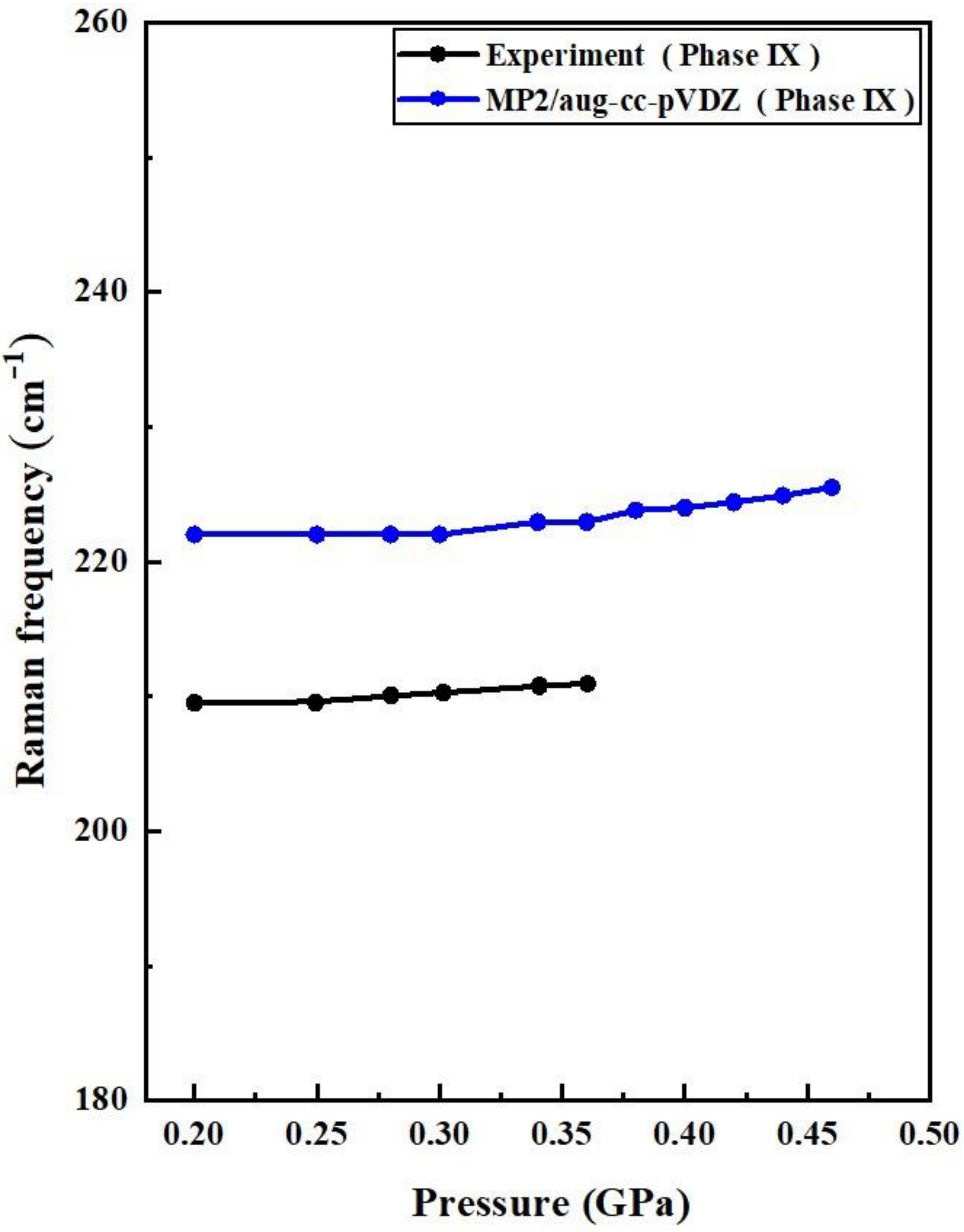

Figure S3. The Raman bands of ice phase IX in the low frequency region under different pressures. The black dots denote the experimental data that are taken from Minceva-Sukarova et al. [13].

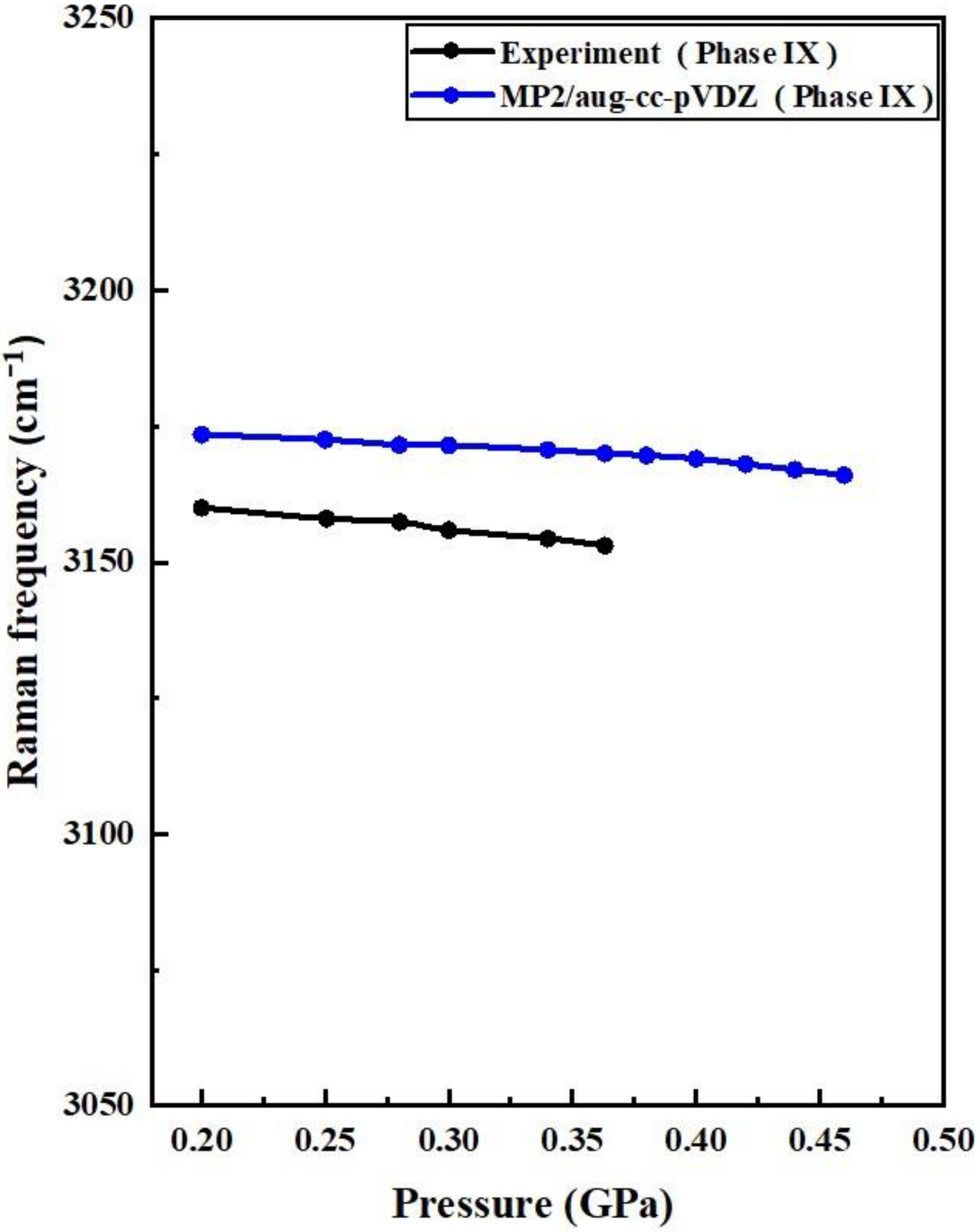

Figure S4. The pressure dependence of the Raman bands of ice IX in the high frequency region as a function of pressure. The blue and black curves represent the calculated and experimental [3] results, respectively.

2. Ice phase XIII

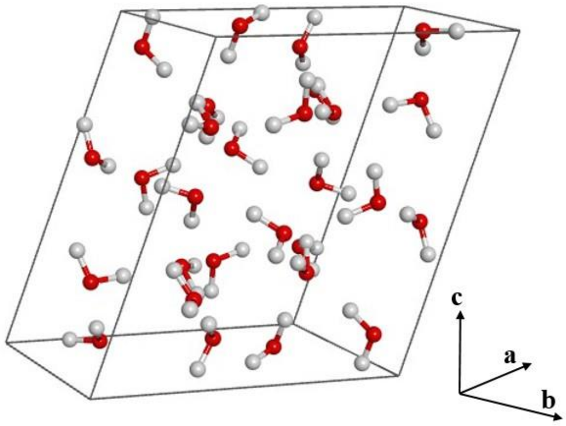

Figure S5. The unit cell (containing 28 water molecules) of ice XIII, which is a proton-ordered and monoclinic structure with the space group of $P2_1/a$.

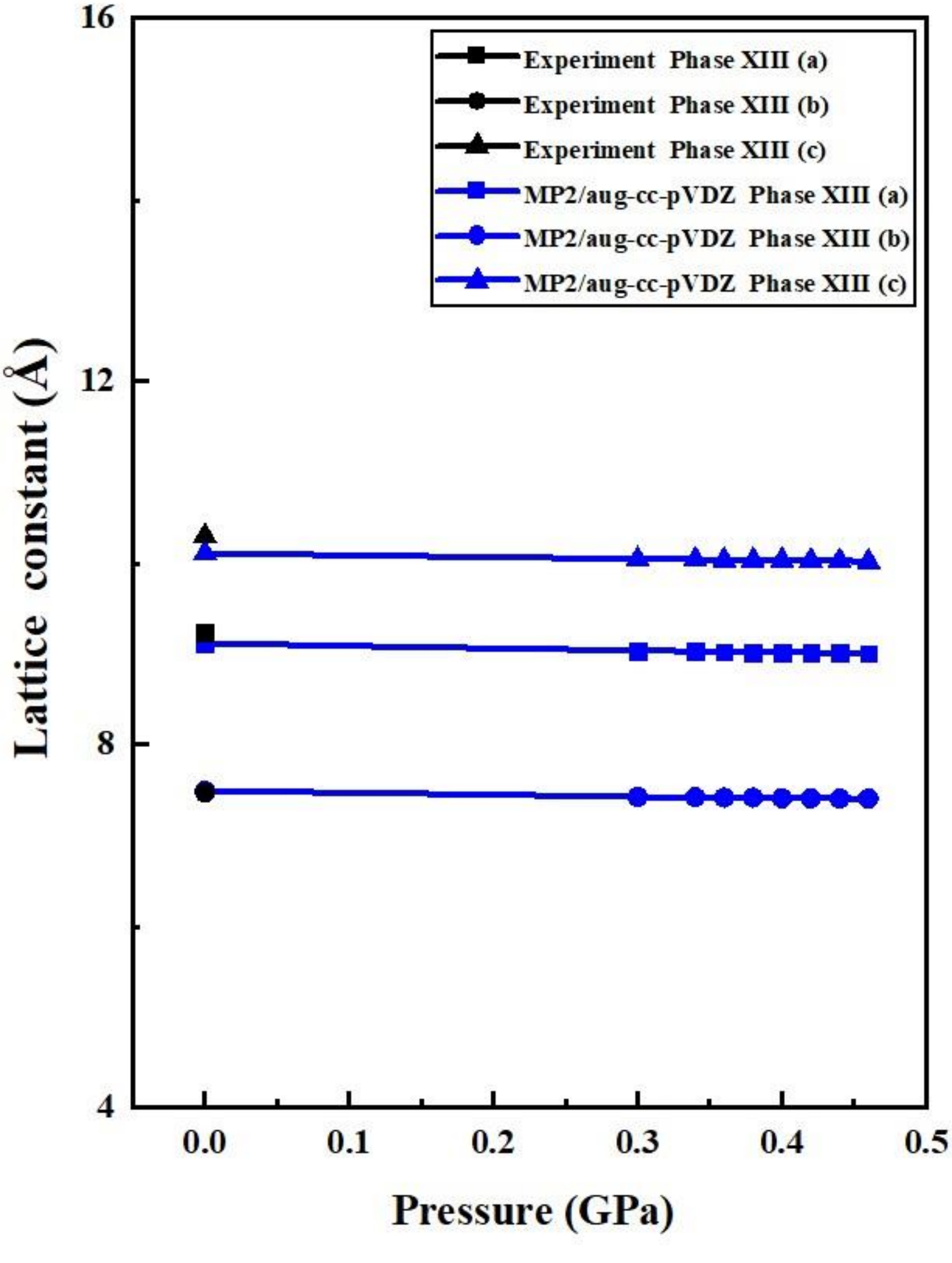

Figure S6. Comparison of the calculated and observed lattice constants of ice XIII, based on the EE-GMF-MP2/aug-cc-pVDZ method. The experimental data are taken from the work by Salzmann et al. [4].

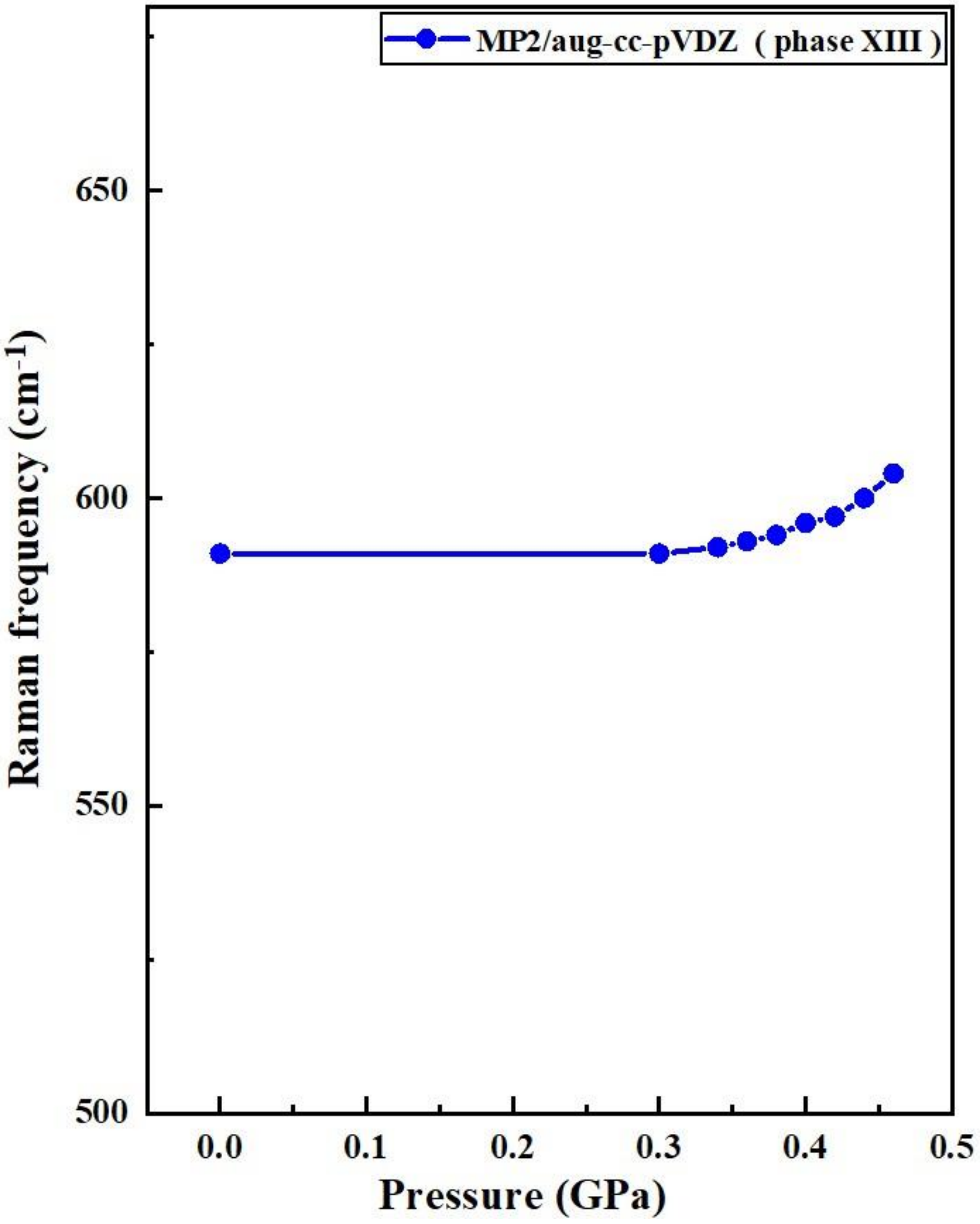

Figure S7. The Raman bands of ice XIII in the low frequency region under different pressures, calculated by the EE-GMF-MP2/aug-cc-pVDZ method.

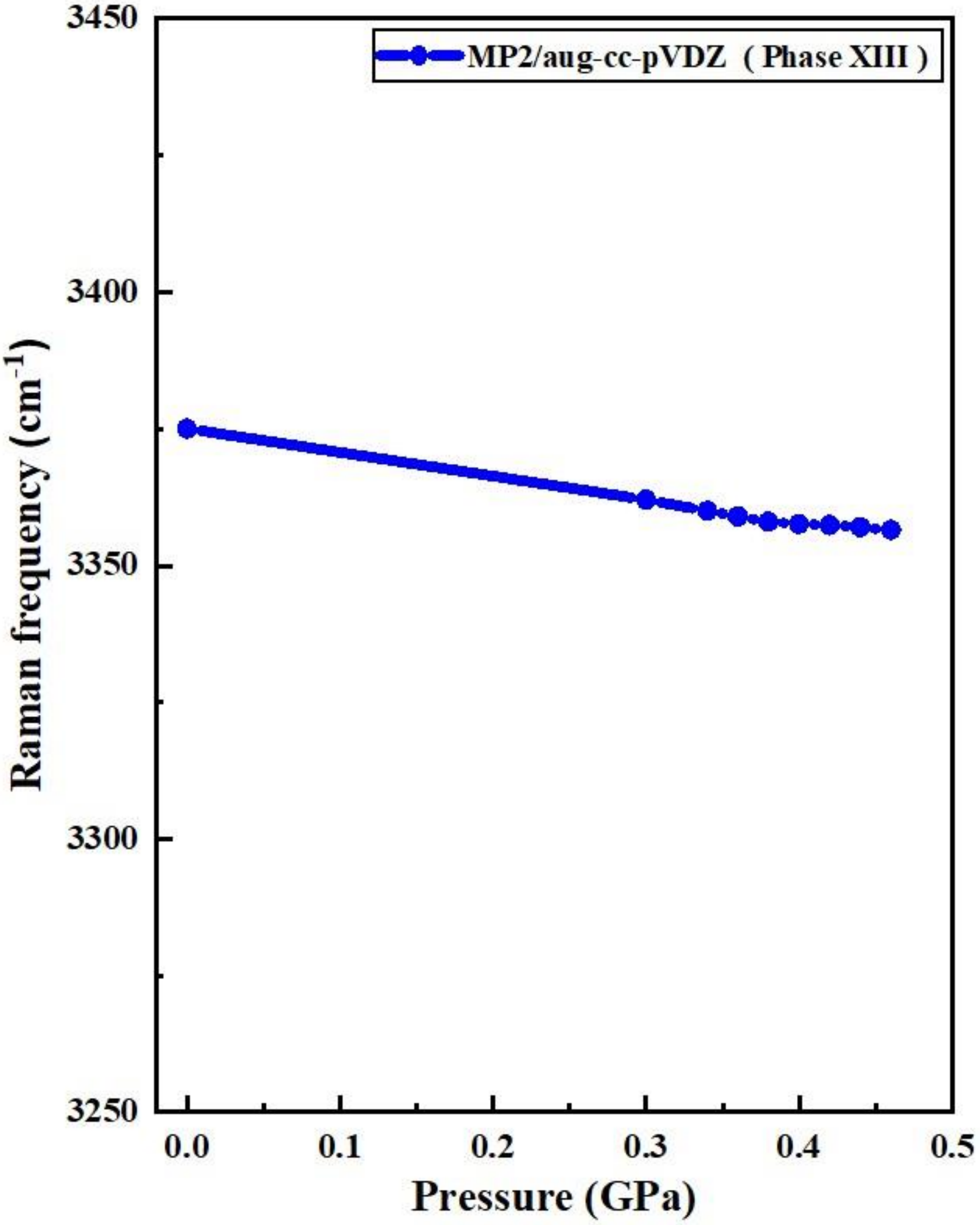

Figure S8. The Raman bands of ice phase XIII in high frequency region under different pressures, calculated by the EE-GMF-MP2/aug-cc-pVDZ method.

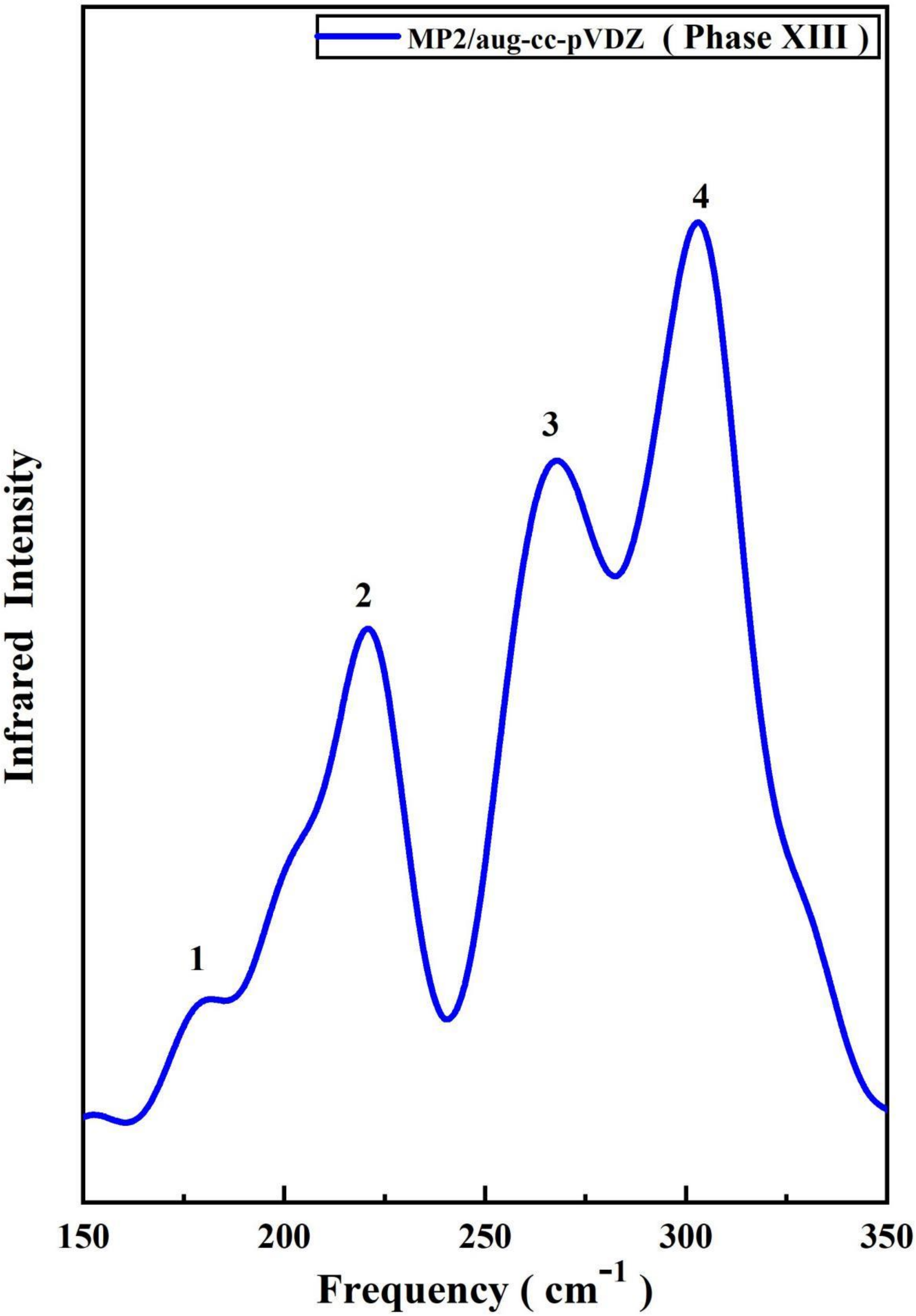

Figure S9. The calculated IR spectrum of ice phase XIII under ambient pressure.

Table S1. Average computational time (in hours) of EE-GMF at the MP2/aug-cc-pVDZ level. All QM calculations were performed using the Gaussian09 program[14].

	Crystal structure optimization	Gibbs free energy calculation
Timings for phase IX	55	12
Timings for phase XIII	74	17

References:

1. J. D. Londono, W. F. Kuhs and J. L. Finney, Neutron diffraction studies of ices III and IX on under-pressure and recovered samples, *J. Chem. Phys*., 1993, **98**, 4878-4888.

2. C. Knight and S. J. Singer, A reexamination of the ice III/IX hydrogen bond ordering phase transition, *J. Chem. Phys*., 2006, **125**, 064506.

3. S. J. La Placa, W. C. Hamilton, B. Kamb and A. Prakash, On a nearly proton-ordered structure for ice IX, *J. Chem. Phys*., 1973, **58**, 567-580.

4. C. G. Salzmann, P. G. Radaelli, A. Hallbrucker, E. Mayer and J. L. Finney, The Preparation and Structures of Hydrogen Ordered Phases of Ice, *Science*, 2006, **311**, 1758.

5. C. G. Salzmann, P. G. Radaelli, E. Mayer and J. L. Finney, Ice XV: A New Thermodynamically Stable Phase of Ice, *Phys. Rev. Lett*., 2009, **103**, 105701.

6. Y. Wang, H. X. Zhang, X. Yang, S. Q. Jiang and A. F. Goncharov, Kinetic boundaries and phase transformations of ice i at high pressure, *J. Chem. Phys*., 2018, **148**, 044508.

7. J. E. Bertie and B. F. Francis, Raman spectra of the O–H and O–D stretching Vibrations of ices II and IX to 25 °K at atmospheric pressure, *J. Chem. Phys*., 1980. **72**. 2213.

8. J. E. Bertie and B. F. Francis, Raman spectra of ices II and IX above 35 K at atmospheric pressure: Translational and rotational vibrations, *J. Chem. Phys*., 1982, **77**, 1-15.

9. C. G. Salzmann, A. Hallbrucker, J. L. Finneyc and E. Mayer, Raman spectroscopic study of hydrogen ordered ice XIII and of its reversible phase transition to disordered ice V, *Phys. Chem. Chem. Phys*., 2006, **8**, 3088-3093.

10. Q. N. Lu, X. He, W.X. Hu, X. J. Chen and J.F. Liu, Stability, vibrations, and diffusion of hydrogen gas in clathrate hydrates: insights from ab initio calculations on condensed-phase crystalline structures. *J. Phys. Chem. C*, 2019, **123**, 12052-12061.

11. W. J. Hua, T. Fang, J. G. Yu and S. H. Li, Geometry optimizations and vibrational

spectra of large molecules from a generalized energy-based fragmentation approach. *J. Phys. Chem. A,* 2008, **112**, 10864-10872.

12. X. He, O. Sode, S. S. Xantheas and S. Hirata, Second-order many-body perturbation study of ice Ih. *J. Chem. Phys.,* 2012, **137**, 204505.

13. B . Minceva-Sukarova, W. F. Sherman and G. R. Wilkinson, The Raman spectra of ice (Ih, II, III, V, VI and IX) as functions of pressure and temperature, *J. Phys. C: Solid State Phys*., 1984, **17**, 5833.

14. M. J. Frisch, G. W. Trucks, H. B. Schlegel, G. E. Scuseria, M. A. Robb, J. R. Cheeseman, G. Scalmani, V. Barone, B. Mennucci, G. A. Petersson, H. Nakatsuji, M. Caricato, X. Li, H. P. Hratchian, A. F. Izmaylov, J. Bloino, G. Zheng, J. L. Sonnenberg, M. Hada, M. Ehara, K. Toyota, R. Fukuda, J. Hasegawa, M. Ishida, T. Nakajima, Y. Honda, O. Kitao, H. Nakai, T. Vreven, J. A. Montgomery Jr., J. E. Peralta, F. Ogliaro, M. J. Bearpark, J. Heyd, E. N. Brothers, K. N. Kudin, V. N. Staroverov, R. Kobayashi, J. Normand, K. Raghavachari, A. P. Rendell, J. C. Burant, S. S. Iyengar, J. Tomasi, M. Cossi, N. Rega, N. J. Millam, M. Klene, J. E. Knox, J. B. Cross, V. Bakken, C. Adamo, J. Jaramillo, R. Gomperts, R. E. Stratmann, O. Yazyev, A. J. Austin, R. Cammi, C. Pomelli, J. W. Ochterski, R. L. Martin, K. Morokuma, V. G. Zakrzewski, G. A. Voth, P. Salvador, J. J. Dannenberg, S. Dapprich, A. D.Daniels,Ö.Farkas, J. B. Foresman, J. V. Ortiz, J. Cioslowski and D. J. Fox, Gaussian 09, Gaussian Inc., Wallingford, CT, US.
